# Bisphenols exposure and non-alcoholic fatty liver disease: from environmental trigger to molecular pathogenesis

**DOI:** 10.3389/fendo.2025.1606654

**Published:** 2025-05-22

**Authors:** Chang-Lei Li, Zhi-Yuan Yao, Yin-Feng Zhang, Xiao-Tong Cui, Ao Sun, Jing-Yu Cao, Zu-Sen Wang

**Affiliations:** ^1^ Department of Hepatobiliary and Pancreatic Surgery, The Affiliated Hospital of Qingdao University, Qingdao, Shandong, China; ^2^ Department of Thoracic Surgery, The Affiliated Hospital of Qingdao University, Qingdao, Shandong, China; ^3^ Institute for Translational Medicine, The Affiliated Hospital of Qingdao University, College of Medicine, Qingdao University, Qingdao, China

**Keywords:** non-alcoholic fatty liver disease, bisphenols, environmental epidemiology, molecular pathogenesis, bisphenol A

## Abstract

Bisphenols (BPs), including bisphenol A (BPA) and its substitutes (BPS, BPF), are ubiquitous environmental contaminants with emerging links to metabolic disorders. This review synthesizes current evidence on the role of BP exposure in the pathogenesis of non-alcoholic fatty liver disease (NAFLD), a global health crisis affecting 25% of adults worldwide. Epidemiological studies reveal significant positive associations between urinary/serum BP levels and NAFLD risk, particularly in males, with maternal exposure correlating to transgenerational metabolic dysfunction. Mechanistically, BPs disrupt hepatic lipid homeostasis by activating PPAR-γ and suppressing fatty acid oxidation while concurrently inducing insulin resistance via impaired IRS-1/PI3K/Akt signaling. Oxidative stress, NLRP3 inflammasome activation, and gut-liver axis perturbations further exacerbate steatosis and inflammation. Co-exposure with phthalates or high-fat diets amplifies hepatotoxicity, highlighting synergistic environmental risks. Critically, developmental and sex-specific susceptibility underscores the need for tailored interventions. We propose preventive strategies to mitigate NAFLD progression, including BP avoidance and policy reforms. This work bridges gaps between environmental epidemiology and molecular toxicology, emphasizing BPs as modifiable drivers of metabolic liver disease.

## 1 Introduction

Non-alcoholic fatty liver disease (NAFLD) has emerged as a global health crisis, affecting approximately 25% of the worldwide population, with its progression to non-alcoholic steatohepatitis (NASH) and cirrhosis posing significant clinical challenges (1, 2). While genetic predisposition and metabolic syndromes like obesity are well-established risk factors (3), mounting evidence underscores the critical role of environmental endocrine-disrupting chemicals (EDCs), particularly bisphenols, in modulating NAFLD pathogenesis (4–6). Among these, bisphenol A (BPA) and its analogs (e.g., BPS, BPF) are ubiquitous environmental triggers, leaching from polycarbonate plastics, food packaging, and thermal paper into human ecosystems, resulting in detectable serum levels in >90% of the general population (7).

Recent epidemiological studies reveal a dose-dependent association between urinary BPA levels and NAFLD severity, independent of traditional risk factors like BMI (8). Mechanistically, BPA disrupts hepatic lipid homeostasis through two distinct mechanisms: (1) estrogen receptor (ER) antagonism, impairing lipid oxidation and promoting ectopic fat deposition (9, 10), and (2) NLRP3 inflammasome activation, driving pro-inflammatory cytokine release (e.g., TNF-α, IL-1β, and IL-6) that exacerbates hepatic insulin resistance (11, 12). Notably, emerging data suggest that BPA’s effects extend beyond direct hepatotoxicity, involving gut-liver axis dysregulation—via gut microbiota-derived metabolites like secondary bile acids and trimethylamine-N-oxide (TMAO)—that synergistically potentiates hepatic *de novo* lipogenesiss and oxidative stress (13, 14).

Despite these advances, critical knowledge gaps persist: (1) Temporal Dynamics: Most studies focus on acute BPA exposure, neglecting chronic low-dose effects that mimic real-world scenarios; (2) Epigenetic Modulation: BPA-induced DNA methylation changes in genes regulating lipid metabolism remain underexplored in NAFLD progression; (3) Cumulative Exposures: Synergistic interactions between bisphenols and other environmental stressors (e.g., microplastics, heavy metals) are poorly characterized but may explain geographic disparities in NAFLD prevalence.

**Figure 1:**
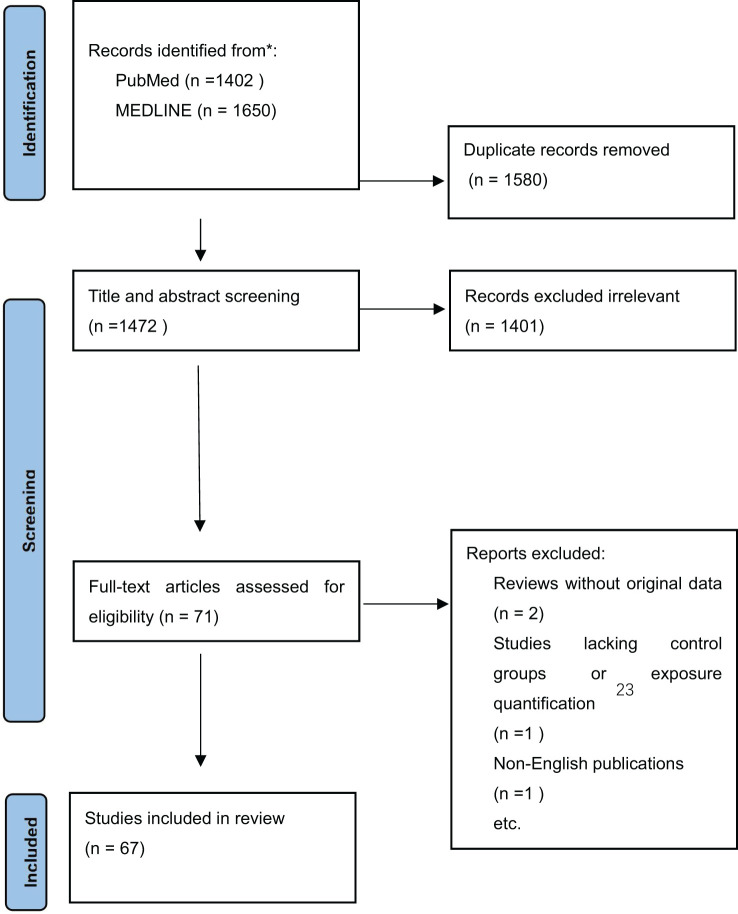
PRISMA flow diagram for study selection.

This review bridges these critical knowledge gaps by systematically integrating epidemiological insights with mechanistic toxicological evidence to elucidate the multifaceted role of BPs in NAFLD pathogenesis. Herein, we conducted rigor methodology in literature selection (**Figure 1**): (1) Systematic Search Protocol Databases: We conducted searches in PubMed and MEDLINE using controlled vocabulary (e.g., MeSH terms: “Bisphenols,” “NAFLD,” “Environmental Exposure”) and free-text keywords (e.g., “endocrine disruptors,” “hepatic steatosis,” “PPAR-γ”). (2) Timeframe: Focused on 2000–2025 to capture modern exposure patterns and molecular mechanistic insights. (3) Inclusion Criteria: Human epidemiological studies (cross-sectional, cohort) and experimental models (rodent, zebrafish, cell lines); Studies reporting quantifiable BP exposure levels (urinary/serum biomarkers); Mechanistic data on lipid metabolism, insulin signaling, or oxidative stress pathways. (4) Exclusion Criteria: Reviews without original data Studies lacking control groups or exposure quantification Non-English publications (to ensure quality interpretation). We synthesize emerging data on chronic low-dose BP exposure effects, emphasizing non-monotonic dose-response relationships and developmental windows of susceptibility. A key focus is on epigenetic reprogramming mechanisms, particularly BP-induced DNA methylation changes in lipid regulatory genes and their transgenerational metabolic consequences. Furthermore, we characterize synergistic hepatotoxicity arising from co-exposures to BPs and other environmental stressors, such as phthalates and high-fat diets, which amplify oxidative stress and gut-liver axis dysfunction. By contextualizing molecular pathways within population-level exposure gradients—including occupational hazards and socioeconomic disparities in BP exposure—this work advances a unified framework linking environmental triggers to clinical NAFLD phenotypes. Finally, we propose evidence-based preventive strategies, from BP-free product alternatives to policy reforms regulating cumulative exposures, while identifying priority research directions for mitigating the global burden of metabolic liver diseases.

## 2 Environmental exposure to bisphenols

### 2.1 Ubiquitous sources and exposure routes

BPA and its analogs (e.g., BPS, BPF and BPAF) are extensively used in polycarbonate plastics, epoxy resins, thermal paper receipts, food packaging, and dental sealants (15–17). For example, BPA-free alternatives like phenol stearic acid-based polyether resins are increasingly adopted in coatings, though their long-term safety remains understudied (18). Non-dietary sources include household dust, personal care products, and occupational settings (e.g., cashiers handling thermal paper receipts).

**Figure 2:**
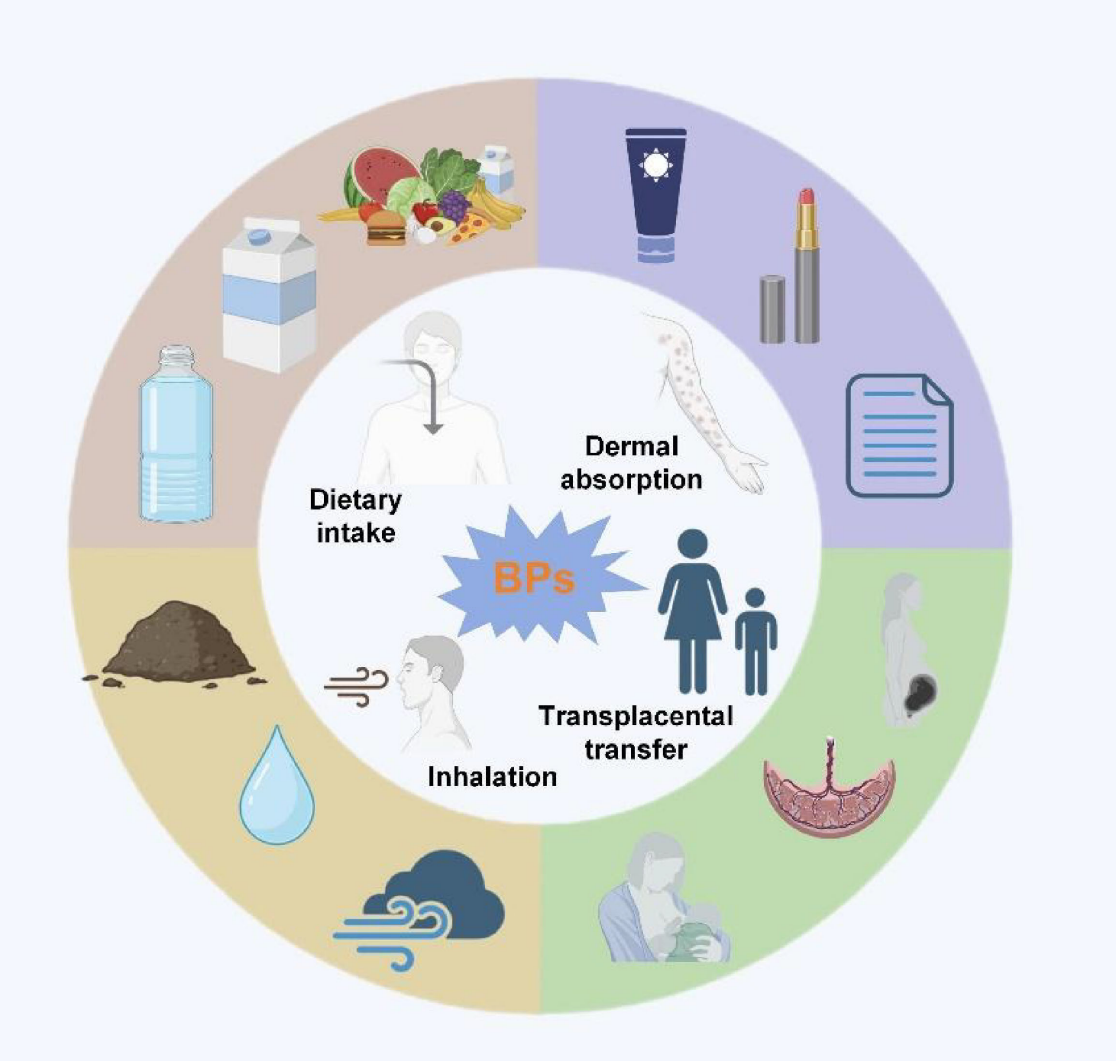
Four common pathways humans can be exposed to BPA and its analogs.

Humans can be exposed to BPA and its analogs by common pathways (summarized in **Figure 2**): (1) Dietary intake: Leaching of BPs from food containers and canned goods into foodstuffs, especially under high-temperature conditions (19); (2) Dermal absorption: direct skin contact with thermal paper (e.g., receipts) or cosmetics containing BP derivatives (20); (3) Inhalation: inhalation of BP-laden dust particles in indoor environments (21) and (4) Transplacental and lactational transfer: maternal exposure leads to fetal and infant exposure via placental circulation and breast milk (22).

### 2.2 Biomarkers and population-level burden

Biomonitoring evidence demonstrates pervasive human exposure to BPs. BPs and their phase II metabolites (e.g., BPA-glucuronide) are routinely detected in human biofluids, with detection rates exceeding 95% in urine, serum, and breast milk samples across global populations (23, 24). Occupational exposure gradients are particularly striking: urinary BP concentrations in cashiers and factory workers handling BP-containing thermal paper or epoxy resins are 3- to 5-fold higher than in the general population, reflecting direct dermal and inhalation exposure pathways.

Concerningly, three vulnerable subpopulations are facing disproportionate risks induced by BPs: Firstly, children exhibit elevated exposure per unit body weight due to developmentally driven behaviors (e.g., frequent hand-to-mouth contact) coupled with immature hepatic detoxification systems that prolong BP half-lives (25, 26); Secondly, low-income communities experience dual burdens: limited access to BP-free alternatives and dependence on processed/packaged foods (primary dietary sources of BP migration from can linings and plastic containers) (27, 28); Moreover, pregnant women represent a critical susceptibility window, as transplacental BP transfer during fetal developmental programming phases may induce epigenetic alterations with lifelong health consequences (29).

### 2.3 Temporal trends and regulatory gaps

The shift from BPA due to regulatory restrictions (e.g., bans on baby bottles) has led to the adoption of structurally similar analogs such as BPS and BPF (30). However, these substitutes exhibit comparable endocrine-disrupting properties, and their long-term toxicological profiles remain poorly characterized. Emerging alternatives like bisphenol AP (BPAP) and bisphenol AF (BPAF) are increasingly detected in environmental samples, yet their safety and health impacts are understudied.

A significant challenge in regulating BPs lies in the lack of harmonized standards. Current regulations focus on individual BPs (e.g., BPA) but fail to address the cumulative effects of co-exposure to multiple analogs. Additionally, biomonitoring efforts are hindered by the short half-lives of BP metabolites (e.g., less than 6 hours for BPA), complicating accurate exposure assessment in epidemiological studies (31). Another critical gap is the insufficient interdisciplinary collaboration among environmental science, toxicology, and public health, which weakens the scientific foundation for risk assessments and regulatory measures. Furthermore, public awareness of BP risks remains limited, highlighting the need for targeted health education initiatives.

Future research should prioritize comprehensive toxicological studies on emerging BP substitutes, particularly their long-term health effects. Policymakers must develop unified regulatory standards for cumulative exposures and improve biomonitoring technologies to assess population-level risks better. Addressing these gaps is essential to mitigate the health impacts of BPs and their analogs.

## 3 Epidemiological evidence linking BPs to NAFLD

A growing body of epidemiological studies has established significant associations between BPs exposure and NAFLD risk across diverse populations. Key findings from large-scale human studies are summarized below (**Table 1**).

**Table 1:** Epidemiological evidence linking bisphenol exposure to NAFLD risk.

Study (Year)	Population/Cohort	Exposure Assessment	Key Findings	Limitations
Verstraete et al. (32)	944 US adolescents (NHANES)	Urinary BPA quartiles	Q2 vs Q1: OR=4.23 (1.44-12.41) Stronger in Hispanics (OR=6.12)	Cross-sectional design ALT unaffected
Kim et al. (33)	7,605 US adults (NHANES)	Urinary BPA quartiles	Q3: OR=1.69 (1.39-2.04) Q4: OR=1.44 (1.19-1.76)	Hepatic Steatosis Index-based diagnosis
An et al. (34)	3,476 Korean adults (KoNEHS)	Urinary BPA quartiles	Q4: adj.OR=1.32 (1.03-1.70) Linear dose-response	Single urinary measurement
Peng et al. (8)	960 US adults (NHANES)	Urinary BPA/BPS tertiles	BPA: OR=1.42 (1.11-1.82) 23.1% insulin mediation	HSI diagnostic threshold
Liang et al. (35)	Chinese + US cohorts	Serum/urinary BPS	Chinese cohort: OR=3.98 (3.42-4.63) Consistent cross-population effects	Potential residual confounding

### 3.1 Dose-response relationships in adolescent and adult populations

In U.S. cohorts, previously in 2018, Sofia et al. utilized National Health and Nutrition Examination Survey (NHANES) data (2003-2010) on 12-19-year-old adolescents. They incorporated a total of 944 urinary BPA and fasting laboratory tests from a total of 7168 adolescents. They calculated that the risk of suspected NAFLD was increased in the second quartile of BPA levels (1.4-2.7 ng/mL) when compared to the first (< 1.4 ng/mL) (Odds Ratio (OR) 4.23, 95% Confidence Interval (CI) 1.44-12.41) (32). Interestingly, the association was stronger in Hispanics (n = 344) with BPA levels in the second (OR 6.12, 95% CI 1.62-23.15) quartile and when limiting the analyses to overweight/obese adolescents (n = 332), in the second (OR 5.56, 95% CI 1.28-24.06) and fourth BPA quartiles (OR 6.85, 95% CI: 1.02-46.22) compared to the first quartile. However, BPA levels were not associated with ALT elevation, indicating that laboratory indexes could not estimate BPA-induced hepatoxicity.

Apart from teenagers, in 2019, Donghee and colleagues conducted a cross-sectional analysis of data from the NHANES database (2005 - 2014) focusing on in the US adults (33). Among the initial 7605 participants (with a mean age of 47 years and 48.4% being male), a correlation was found between the prevalence of NAFLD and abnormally elevated ALT levels and urinary BPA levels (P < 0.05). Compared to the reference group with the lowest urinary BPA levels, individuals in the third and fourth quartiles had an 81% and 53% higher likelihood of developing NAFLD, as defined by the hepatic steatosis index (HIS). In a multivariate model, the ORs for NAFLD in the third and fourth quartiles were 1.69 (95% CI (36): 1.39 - 2.04) and 1.44 (95% CI: 1.19-1.76) respectively (p< 0.001).

Similarly to U.S. cohorts, Yang’s research team consistently conducted the Korean National Environmental Health Survey (KoNEHS, 2015-2017) involving 3,476 adults (1,474 men and 2,002 women) (34). In their research, abdominal ultrasonography (hyperechoic liver parenchyma and vessel blurring) was considered as the gold-standard imaging criterion (37). Their findings illustrated a linear association between urinary BPA concentrations and NAFLD risk. In a univariate analysis, the OR for NAFLD in the highest quartile of urinary BPA levels was found to be 1.47 [95% CI: 1.11-1.94] compared to the lowest quartile. After adjusting for covariates, the ORs for NAFLD in the third and fourth quartiles were determined to be 1.31 [95% CI: 1.03-1.67] and 1.32 [95% CI: 1.03-1.70], respectively.

Similarly, a cross-sectional study (n=960 U.S. adults) using the Hepatic Steatosis Index (HSI) [HSI > 36 was used to predict NAFLD (38)] identified 1.42-fold (BPA, 95% CI: 1.11–1.82) and 1.31-fold (BPS, 95% CI: 1.02–1.68) elevated NAFLD risks per unit increase in urinary BP concentrations. Mediation analyses identified insulin resistance pathways as accounting for 18.7-23.1% of the observed risk elevation (8). Similarly, Chinese cohorts showed striking serum BPS-associated NAFLD risks (OR=3.16 per unit increase, 95% CI: 2.81–3.55) (35).

### 3.2 Critical vulnerable subpopulations

BPs induced the development of NAFLD partially due to their sex-specific effects. Meta-analyses highlight stronger BP-NAFLD associations in males, potentially linked to androgen receptor crosstalk with estrogenic BP metabolites (8, 33, 39, 40). Concerningly, here exists socioeconomic disparities. Low-income populations face dual exposure risks due to greater dependence on processed/packaged foods (primary sources of BP migration) coupled with limited availability of BP-free alternatives (27, 28).

### 3.3 Methodological limitations and unresolved questions

Most studies are cross-sectional, precluding causal conclusions. Longitudinal data tracking BP exposure and liver outcomes (e.g., fibrosis, cirrhosis) over decades are urgently needed. Additionally, short half-lives of BP metabolites (e.g., BPA-glucuronide: <6 hours) complicate accurate exposure assessment, necessitating repeated biomonitoring in future studies (31). Epidemiological data on BPF, BPAF, and other substitutes are scarce despite their rising environmental prevalence (30).

Collectively, according to epidemiological research, exposure to BPs, especially BPA and BPS, is demonstrated to be related to increased risk of NAFLD for adolescents and adults. Though lots of evidence supports the suggestive relationship, unsolved limitations still exist. Most existing studies are cross-sectional, limiting causal inference between BP exposure and NAFLD progression. Thus, longitudinal data tracking BP levels and liver health outcomes over decades are critically needed, especially the risk of liver fibrosis, chronic hepatitis, and even liver cancer. Also, metabolites (e.g., BPA-glucuronide) exhibit short half-lives, complicating exposure assessment. Additionally, co-exposure to other endocrine disruptors (e.g., phthalates) may confound observed associations. While BPA and BPS have been partially characterized in the risk of NAFLD, epidemiological data on BPF, BPAF, and other substitutes remain scarce despite their increasing environmental prevalence.

## 4 Molecular mechanisms of BPs in NAFLD pathogenesis

**Figure 3:**
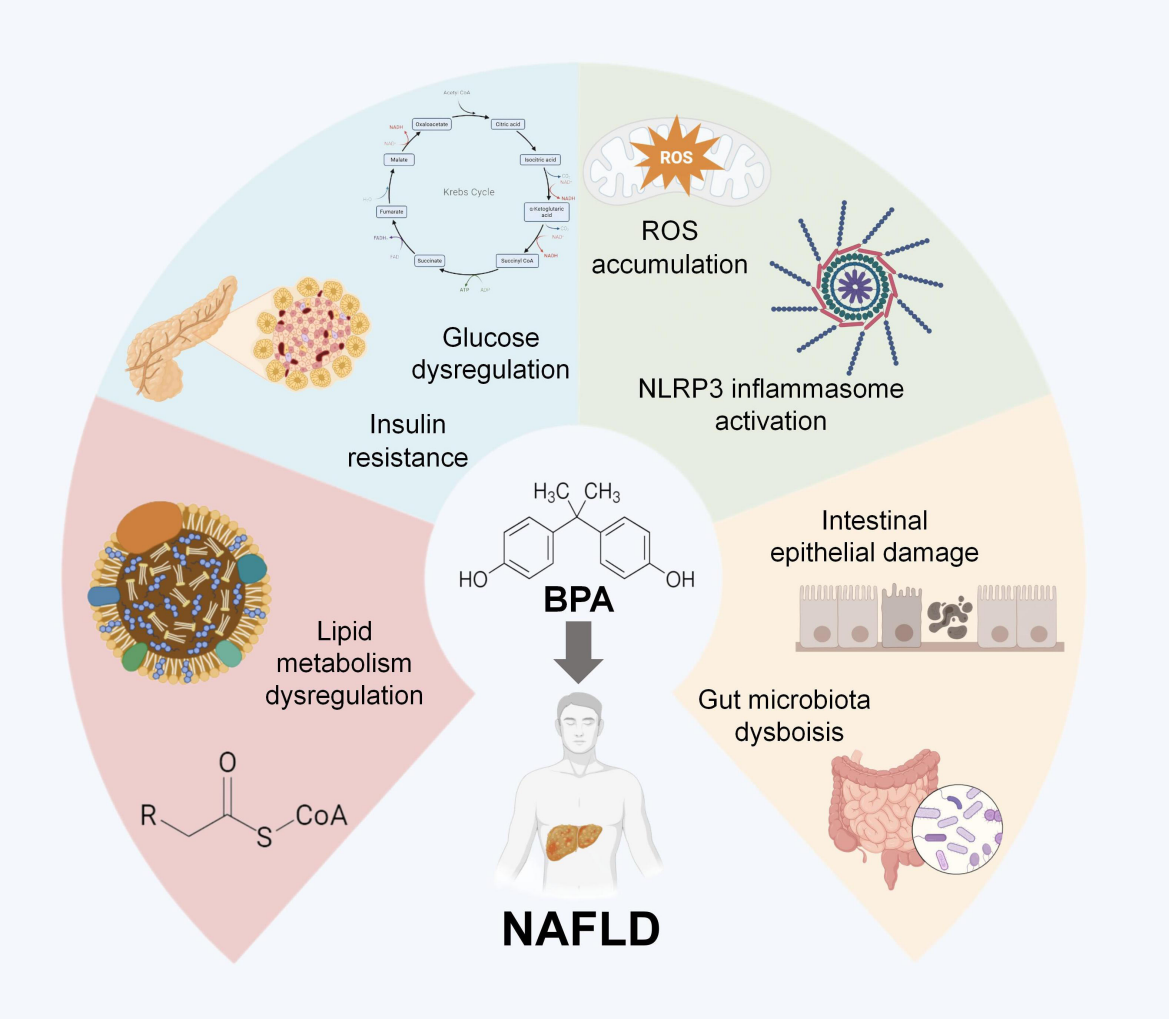
Four major mechanisms BPA in the pathogenesis and development of NAFLD: lipid metabolism disruption, insulin resistance and glucose dysregulation, oxidative stress, and gut-liver axis perturbation.

In this part, we summarize the predominant roles of BPs in the pathogenesis and development of NAFLD: lipid metabolism disruption, insulin resistance and glucose dysregulation, oxidative stress, and even gut-liver axis perturbation (**Figure 3**, **Table 2**).

**Table 2:** Molecular mechanisms of BPA-Induced NAFLD pathogenesis.

Pathway	Key Targets	Experimental Model	Functional Consequences	Refs
Lipid Metabolism	PPARγ, SREBP-1c, SCD1, APOD	CD1 mice (28-day exposure)	↑Hepatic TG (40%↑), ↑Cholesterol esters	(44, 47)
Insulin Resistance	IRS-1/PI3K/Akt	3T3-L1 adipocytes + C57BL/6 mice	↓Glucose uptake (35%↓), ↑ HOMA-IR	(46, 48)
Oxidative Stress	NLRP3, OGT, Nrf2	Zebrafish + HepG2 cells	↑ ROS (2.8-fold), ↑ IL-1β (4.5-fold)	(49, 51)
Epigenetic Regulation	miR-192, SREBF1	Perinatal mouse exposure	↓Global m6A (32%↓), Hypomethylation at Srebp-1c	(50, 52)
Gut-Liver Axis	TMAO, Bile acids	Human microbiome studies	↑F/B ratio (1.7-fold), ↑ LPS translocation	(13, 14)

### 4.1 Disruption of lipid metabolism

Lipid metabolism disruption plays a causal role in the development and progression of NAFLD (41–43). Initially, Marmugi et al. conducted a 28-day oral exposure study in male CD1 mice with BPA doses ranging from 0, 5, 50, 500, and 5,000 μg/kg/day, revealing low-dose-specific hepatic effects, particularly on lipid synthesis genes. Human daily intake estimates for BPA range from 0.01–0.1 μg/kg/day in the general population, with occupational exposures reaching up to 1–10 μg/kg/day. Notably, the No Observed Adverse Effect Level (NOAEL) for BPA in rodents is 5 mg/kg/day, far exceeding the highest experimental dose (5,000 μg/kg/day = 5 mg/kg/day).

BPA-induced nonmonotonic dose-response patterns in *de novo* lipogenesis genes (Acc, Fasn, Scd1) and regulatory transcription factors (LXR, SREBP-1c, ChREBP), with more substantial impacts at lower doses. Hepatic cholesterol esters and triglyceride accumulation confirmed enhanced fatty acid biosynthesis (44). Detectable BPA plasma levels have also been observed in Savastano’s research. The robust association between BPA and waist circumference, components of metabolic syndrome, along with inflammatory markers (43) (insulin resistance index, plasma monocyte chemoattractant protein 1, interleukin-6 and tumor necrosis factor-alpha, further substantiates BPA’s involvement in visceral obesity-associated low-grade chronic inflammation (45).

Then, Wang’s group employed an early-life BPA exposure model and further assessed the impacts of BPA exposure on lipid homeostasis (46). *In vitro*, 3T3-L1 adipocytes exposed to 30 μM BPA for 48 hours showed a 40% increase in lipid accumulation (p<0.01, oil red O quantification). *In vivo*, male C57BL/6 mice receiving 0.5 mg/kg/day BPA via oral gavage for 8 weeks developed hepatic steatosis, with liver triglycerides elevated by 80% and 30% (low dose and high dose, respectively) compared to controls (p<0.001, p<0.01). RNA-Seq revealed BPA-altered biological processes, including glycosphingolipid biosynthesis, adipocyte lipolysis regulation, PPAR signaling, and fatty acid metabolism in preadipocytes. Notably, adipose tissue and liver showed significant upregulation of SCD1 and APOD downregulation (p<0.01). Mice exposed to 50 or 500 μg/kg/day BPA for 8 weeks developed NAFLD features, confirming dose-dependent metabolic disruption (47). RNA-seq revealed significant expression reversals in lipid-related genes (SCD1, APOD, ANGPT4, PPARβ, LPL, G0S2) between BPA-exposed and recovery groups, particularly SCD1/APOD (p<0.01). BPA exposure markedly reduced APOD protein (p<0.01), which rebounded post-exposure. APOD overexpression suppressed TG accumulation in AML12 cells, demonstrating its critical role in mitigating BPA-induced hepatic metabolic dysfunction significantly in PPAR-related pathway. Not only by PPARβ, inhibition of PPARγ also participant in BPA (1 μg/kg/day)-induced liver lipid accumulation (48). Dysregulation of lipid-regulating factors were detected in hepatic tissue, with pharmacological inhibition of PPARγ ameliorating gestational BPA exposure-induced hepatic steatosis. Furthermore, a male-specific reduction was observed in HNF1b protein levels in offspring. These findings indicate APOD upregulation repairs BPA-mediated damage, highlighting the need to evaluate BPA exposure risks in chronic liver diseases. Furthermore, BPA (1, 2, and 4 μM for 48 h) disrupts lipid metabolism and triggers pyroptosis by upregulating O-GlcNAc transferase (OGT) in HepG2 cell line (49). NLRP3 directly interacts with OGT, with elevated OGT levels enhancing NLRP3 protein stability. Specifically, BPA enhances OGT-mediated O-GlcNAcylation to stabilize NLRP3, accelerating NAFLD progression *in vitro* models.

More concerningly, the perinatal and peripubertal exposure to BPA should be paid more attention. In 2017, Slitt et al. exposed pregnant mice to 25μg/kg/day BPA from gestation through lactation. Offspring showed persistent fat accumulation via lipogenic gene hypomethylation and elevated hepatic Nrf2 recruitment to Srebp-1c promoters. More epidemiological research should focus on perinatal women, babies, and children (50).

Apart from the mouse model, they also utilized zebrafish to strengthen the assumption (51). Histopathological observation and physiological and biochemical indicators revealed that BPA and TCS (200 mg/L for 90d) exposure led to hepatic fat accumulation in acute and chronic scenarios. RNA-Seq analysis showed that TCS disrupted multiple physiological processes, including drug metabolism, sucrose metabolism, fat metabolism, and bile secretion. The dysregulation of lipid metabolism-related genes indicated that liver steatosis in zebrafish exposed to TCS and BPA resulted from increased fatty acid synthetase, uptake, and suppression of β-oxidation. EDC exposure caused a decrease in global m6A levels and abnormal expression of m6A modulators in larvae.

Apart from protein-coding genes, a classic microRNA—miR-192 also participates in the process of BPA-induced NAFLD (52). Lin et al. showed that after 90-day 50μg/kg/day of BPA by oral gavage, male post-weaning C57BL/6 mice displayed a NAFLD-like phenotype. BPA-induced hepatic steatosis in mouse/HepG2 models correlated with miR-192 downregulation and SREBF1-mediated lipogenic activation. Impaired DROSHA processing reduced miR-192, which directly targeted SREBF1’s 3’UTR. Also, miR-192 overexpression reversed BPA-induced lipid dysregulation by suppressing SREBF1.

Unlike BPA, BPS induced NAFLD through other critical pathways, as Gu’s group reported. BPS activates PPARα-mediated EP300 upregulation, facilitating its nuclear-to-cytoplasmic translocation. This induces Raptor acetylation, triggering mTORC1 activation, and impairs autophagic flux and hepatic lipid metabolism. EP300 knockdown attenuated Raptor acetylation and restored autophagy, identifying EP300 as a key mediator in BPS-induced NAFLD pathogenesis. These findings reveal environmental pollutant-driven metabolic dysregulation mechanisms. Moreover, Qin et al. probed into this fascinating phenomenon through long-term BPS exposure to zebrafish (53). A 120-day BPS exposure induced hepatic steatohepatitis in zebrafish by elevating AST/ALT levels, lipid accumulation (TAG/cholesterol), and fibrosis via PERK-ATF4a UPR pathway activation. While 30-day lipid deposition reversed post-depuration, prolonged exposure upregulated lipogenic genes (srebp1, acc, fasn, and elovl6), triggered ERS-mediated autophagy (atg3, lc3) and inflammation (il1b, tnfα). Findings reveal BPS-driven metabolic disruption in NAFLD pathogenesis through ERS cascades.

Based on Fan et al.’s research, BPF also has the potential for NAFLD risk. Integrating multi-omics methods, they found that BPF exposure (2 mg/kg/day for male and 5 mg/kg/day for female) contributed to changes in hepatic transcriptome, metabolome, and chromatin-accessible regions enriched for binding sites of transcription factors in the bZIP family (54). These alterations were enriched with pathways integral to the ERS and NAFLD, which relied on bZIP family transcription factors. Intriguingly, Drp1 inhibition via Mdivi-1 or gene silencing suppressed mitochondrial fission, alleviating BPF-induced hepatic lipid deposition. Mitochondrial dynamics imbalance mediated this process, as Drp1 blockade reversed fission, restored mitochondrial metabolism, and reduced ROS overproduction - key drivers of lipid accumulation (55). This study identifies Drp1-mediated mitochondrial damage as critical in BPF-triggered NAFLD-like pathology, suggesting mitochondrial-targeted therapies as potential interventions. Liu’s group previously also described a similar phenomenon (56). Interestingly, they deciphered the glycerophospholipid metabolic pathway, which was the most pronounced in BPF-induced disturbance of lipid metabolism. This event revealed a significant mechanism and provided novel intervention strategies for BPF-induced NAFLD-like changes.

### 4.2 Insulin resistance and glucose dysregulation

Insulin resistance and glucose metabolism dysregulation are pivotal risk factors for NAFLD (57, 58). Interestingly, glucose can easily be disrupted by BPs, which might mediate the process of NAFLD development reported by a population-based, cross-sectional study (8). However, the underlying mechanism is complex and includes novel pathways, though BPs have been verified to contribute to insulin resistance by mimicking the effects of strong binders, such as estrogens (59–61). Previously in 2012, Wang et al. conducted a community-based study to dissect the association between BPA exposure’s dose-response association with obesity and insulin resistance in 3390 Shanghai adults (≥40y, mainly middle-aged and elderly Chinese adults) (62). The highest BPA quartile participants showed increased risks of generalized obesity (OR=1.50, 95%CI=1.15-1.97), abdominal obesity (1.28;1.03-1.60), and insulin resistance (1.37;1.06-1.77). Notably, non-overweight individuals (BMI<24kg/m²) demonstrated 94% higher insulin resistance risk (1.94;1.20-3.14) in the top BPA quartile, an association absent in overweight subjects. Long et al.’s study demonstrated that hepatic metabolic analysis revealed BPA-exposed offspring developed hepatic steatosis in both sexes. Male-specific lipid accumulation, along with glucose dysregulation, was observed (48). Sex-dependent HNF1b downregulation occurred in males, suggesting gender-specific metabolic vulnerability.

Similarly, Ji et al. C57BL/6 fed mice with BPA (1-250 μg/kg) for 35 days showed disrupted hepatic fatty acid/glucose metabolism and TCA cycle via metabolomics (high-resolution MS), molecular docking, and enzyme assays. BPA activated nuclear receptor LXR, causing hypoglycemia and impaired liver metabolic functions (63). Thus, disrupted glucose metabolism in BPA-caused NAFLD generally occurs with other abnormal metabolisms.

To investigate the potential relation, Federico et al. analyzed 60 biopsy-confirmed NAFLD patients (with/without T2DM) and 60 healthy controls (64). BPA levels were significantly elevated in NAFLD patients’ plasma/urine vs controls (P<0.0001), particularly in 30 NASH vs 30 simple steatosis cases (P<0.05). A 1-month BPA-free diet reduced plasma BPA (P<0.05) without affecting urinary levels. *In vitro*, 0.05μM BPA exposure under high/low glucose conditions (H-/L-HepG2) for 48h increased HepG2 proliferation vs controls, with lipid peroxidation showing dose-dependent responses. Their findings suggest environmental BPA exposure may influence NAFLD progression regardless of diabetic status. Due to the sample size limitation, this conclusion still needs further research.

Intriguingly, ovariectomized (OVX) HFD-fed females exhibited obesity, glucose intolerance, insulin resistance, and moderate hepatic steatosis, linked to upregulated hepatic lipogenic (Srebf1, Scd1), β-oxidative (Cpt1a), and ER stress (Hspa5, Hyou1) genes (65). BPA exacerbated hepatic steatosis in the OVX HBPA group, elevating lipid/collagen deposition with reduced Mttp mRNA and upregulated β-oxidation (Acox1, Acadvl), mitochondrial uncoupling (Ucp2), ER stress (Hyou1, Atf6), and liver injury (Tgfb1, Casp8) genes. In OVX CBPA, BPA induced mild steatosis, increasing hepatic lipids and lipogenic/ER stress gene (Srebf1, Scd1, Hspa5, Atf6) expression. BPA amplified HFD-driven liver damage without affecting metabolic disruptions.

Not only in mouse models, Shankar’s study in Wistar rats revealed that high-dose BPA (200mg) significantly reduced hepatic insulin receptor and Akt mRNA/protein expression (66). Despite elevated serum insulin and decreased testosterone in the high-dose group, fasting glucose remained stable. Both BPA doses impaired glucose oxidation and glycogen storage, indicating defective insulin signaling disrupts hepatic glucose metabolism without altering baseline glycemia.

In Jeung’s study, BPA and octylphenol (OP) elevate insulin levels in pancreatic β-cells but impair glucose regulation, suggesting disrupted calcium homeostasis. In STZ-induced diabetes models, both chemicals promote β-cell survival yet fail to normalize blood glucose despite hyperinsulinemia (67). They downregulate calcium influx genes (cytosolic/ER Ca²^+^ transport) while upregulating Ca²^+^ efflux pathways, depleting ER calcium stores and triggering ER stress. This ER stress induces insulin resistance, evidenced by reduced GLUT4 and IRS2 expression. Mechanistically, calcium dysregulation by BPA/OP links ER stress to impaired insulin signaling, particularly exacerbating metabolic dysfunction in type 1 diabetes contexts.

Furthermore, the sheep model is also applied to probe into this phenomenon by Muraly et al. (68). Prenatal BPA exposure induced insulin resistance and adipocyte hypertrophy in female offspring. A study on 21-month-old offspring from mothers exposed to 0–5 mg/kg/day BPA revealed non-monotonic dose effects: elevated oxidative stress, hepatic/muscular lipotoxicity, and upregulated aromatase/estrogen receptors in visceral fat. These alterations correlated with tissue-level IR mechanisms, potentially explaining metabolic dysregulation observed in BPA-exposed females.

Geng et al. demonstrated that 5-day 100nM BPA exposure impaired glucose uptake and insulin signaling in HepG2 cells, triggering inflammation, oxidative stress, and JNK/p38 pathway activation. While ERK/NF-κB inhibition showed no effect, blocking JNK/p38 restored metabolic functions (69). Interestingly, curcumin counteracted BPA-induced insulin resistance, but its protective effects were reversed by JNK/p38 activator anisomycin, confirming these pathways’ critical role. However, this therapeutic method warrants further *in vivo* experiments.

### 4.3 Oxidative stress and inflammatory cascades

Oxidative stress and inflammation are involved in the BPs-related liver disease development (70). Previously, in 2016, Sahar et al. utilized 30 male Wistar albino rats (BPA in 50 mg/kg body weight/day, 8 weeks). They concluded that elevated serum hepatic enzymes, hepatic hydroxyproline, and portal collagen deposition evidenced BPA-induced liver fibrosis. It triggered inflammation (↑IL-1β, ↓IL-10), oxidative stress (↑MDA, ↓GSH, suppressed CAT), and apoptosis (↑caspase-3, ↓BCL2+ hepatocytes). BPA upregulated extracellular matrix turnover gene MMP-9 while downregulating its inhibitor TIMP-2, exacerbating fibrotic progression (71). However, they have not dissected the association between BPA exposure and NAFLD, probably due to the inadequate exposure time. Thus, the role of BPA in oxidative stress during fatty liver disease needs further *in vivo* experiments.

Then, Stefania et al. explored BPA and silybin co-effects on H-HepG2 cells under high glucose (72). BPA induced oxidative stress and oxidized steroid hormones into estrogenic/genotoxic metabolites. Silybin counteracted BPA effects by reducing glucose uptake/lipid peroxidation, activating vitamin D3 synthesis, and preventing steroid oxidation. Western blot revealed silybin-modulated p-ERK/ERK and Caspase-3 expression, while Mass spectrometry confirmed altered lipid/steroid profiles. Findings highlight silybin’s protective role against BPA-induced metabolic disruptions in hepatic cells, which can be a potential therapeutic method in the future.

BPA exacerbated HFD-induced hepatic metabolic dysregulation and mitochondrial dysfunction through oxidative stress elevation and antioxidant depletion while synergistically activating TLR4/NF-κB/NLRP3 axis to amplify inflammatory cytokine production and fibrogenesis (73). Accidentally, NLRP3 inflammasome can be activated in the liver of lactating dams after low-dose BPF exposure (74). BPF administration in lactating dams significantly upregulated iNOS and HO-1d expression, triggered activation of NLRP3 inflammasome components (NLRP3, PyCARD, CASP1), and enhanced secretion of proinflammatory cytokines, including IL-1β, IL-18, IFN-γ, and TNF-α. Nowadays, emerging immunological mechanisms now recognized as pivotal contributors to disease pathogenesis encompass functional impairments in innate immunity, adaptive immunity dysregulation, Toll-like receptor (TLR) signaling anomalies, and gut-liver axis homeostasis disruption. The NLRP3 inflammasome, an intracellular multiprotein complex, orchestrates caspase-1-dependent maturation of interleukin-1β (IL-1β) while propagating sterile metabolic inflammation through coordinated pathological cascades (75, 76).

Female sheep were also applied to dissect the underlying mechanism in BPA-induced NAFLD. Interestingly, accordingly to liver-specific pathways included oxidative stress/lipid synthesis. Non coding RNA (ncRNA) alterations occurred in the liver (77 lncRNAs, 14 miRNAs, 127 snoRNAs, 55 snRNAs), correlating with LCORL/MED17/ZNF41 mRNAs. Discriminant analysis identified tissue-specific gene signatures (liver: PECAM/RDH11/ABCA6/miRNAs), linking BPA to mitochondrial dysfunction, oxidative stress, and metabolic dysregulation (77). Accordingly, liver-spleen axis has been verified to involve in NAFLD development. Well-known reasons in explaining these mechanisms include the spleen involvement in immune regulation (78). Furthermore, the spleen-liver axis supports obesity-induced systemic and fatty liver inflammation via MDSC and NKT cell enrichment (79). Apart from inflammation in liver itself, BPA-induced oxidative stress in extraphepatic tissues is potentially important during NAFLD development. According to Shaibi’s study, both sexes of BPA-exposed mice exhibited elevated peripheral monocytes/lymphocytes (80). Adult spleens showed histopathological toxicity: activated germinal centers and apoptotic cells in white pulp, marked eosinophil/lymphocyte infiltration in red pulp. DNA fragmentation via electrophoresis confirmed apoptosis, while elevated malondialdehyde levels indicated oxidative lipid damage in splenic tissues versus controls. Similarly, Cai’s group demonstrated that BPS exposure triggered splenomegaly, pro-inflammatory polarization, and structural remodeling, while provoking lipidomic disruptions within white pulp immune niches, which may offer novel mechanistic insights into bisphenol-mediated multiorgan toxicity (81).

Thus, oxidative stress and inflammation contribute to the pathogenesis of BPA-caused NAFLD and related phenotypes.

### 4.4 Gut-liver axis perturbation

Microbial metabolites and the “gut-liver” axis are pivotal in liver diseases, including NAFLD (82). Exposomics is perceived to probe into the field of liver pathogenesis. Polyaromatic hydrocarbons, especially the BPA and its alternatives, commonly disturb the liver homeostasis via the “gut-liver” axis (83, 84). Male CD-1 mice fed 50 μg/kg/day BPA for 24 weeks developed hepatic steatosis with reduced gut microbiota diversity. BPA increased Proteobacteria while decreasing Akkermansia abundance, impairing intestinal barrier function (ZO-1/occludin decreased), and elevating endotoxin. This activated hepatic TLR4/NF-κB pathway, upregulating IL-1β, IL-18, TNF-α, and IL-6. Findings suggest BPA-induced steatosis links to gut dysbiosis-mediated endotoxemia and TLR4/NF-κB-driven inflammation (14). This initial conclusion was consistent with Feng’s study, reporting that the relative abundance of Proteobacteria and Firmicutes/Bacteroidetes ratio was increased in BPA-fed mice, and this alteration was reversed by curcumin treatment (13).

Similarly, in Wang et al.’s research, both male and female rats were exposed to BPA (300 mg/kg) by oral gavage for 60 consecutive days (85). The male-BPA group showed significant ALT, TG, TC, and LDL alterations; females exhibited GLB, IBIL, ALP, HDL, and Cr changes. BPA reduced gut microbiota diversity and decreased both sexes’ colon SCFAs (caproic, isobutyric, and isovaleric acids). Serum metabolomics revealed BPA-modulated bile acids, amino acids, hormones, and lipids. In males, immune markers IL-6, IL-23, and TGF-β increased, indicating systemic immune disruption. These findings demonstrate BPA’s sex-specific metabolic interference and gut-microbiota-mediated toxicity mechanisms. However, although the histopathological analysis partially presented hepatic impairment, these changes could not meet the criteria of liver steatosis diagnosis. Liao et al. performed a systems biology analysis evaluating liver transcriptomes, gut microbiota, and metabolic phenotypes in mouse offspring exposed to 5 μg/kg/day BPA during gestation (86). Prenatal BPA disrupted hepatic oxidative phosphorylation, PPAR signaling, and fatty acid metabolism genes while inducing sex/age-dependent microbial shifts. Key bacteria (S24-7, Lachnospiraceae) correlated with altered metabolic genes (Acadl, Dgat1) and network drivers (Malat1, Apoa2). This multi-omics integration reveals microbiota-liver interactions potentially mediating cardiometabolic risks from developmental BPA exposure.

Emerging as a prevalent substitute for BPA, BPS exposure induces gut microbiota dysbiosis while triggering obesity, hepatic steatosis, intestinal pathologies, and metabolic dysregulation (87). Notably, robust associations have been established between specific microbial compositional shifts and clinical health parameters in exposed hosts. This evidence positions particular gut microbial signatures as promising BPS exposure risk assessment diagnostic biomarkers (88).

## 5 Sex-specific and developmental susceptibility in mature

### 5.1 Sexual dimorphism in bisphenol-induced metabolic dysregulation

**Figure 4:**
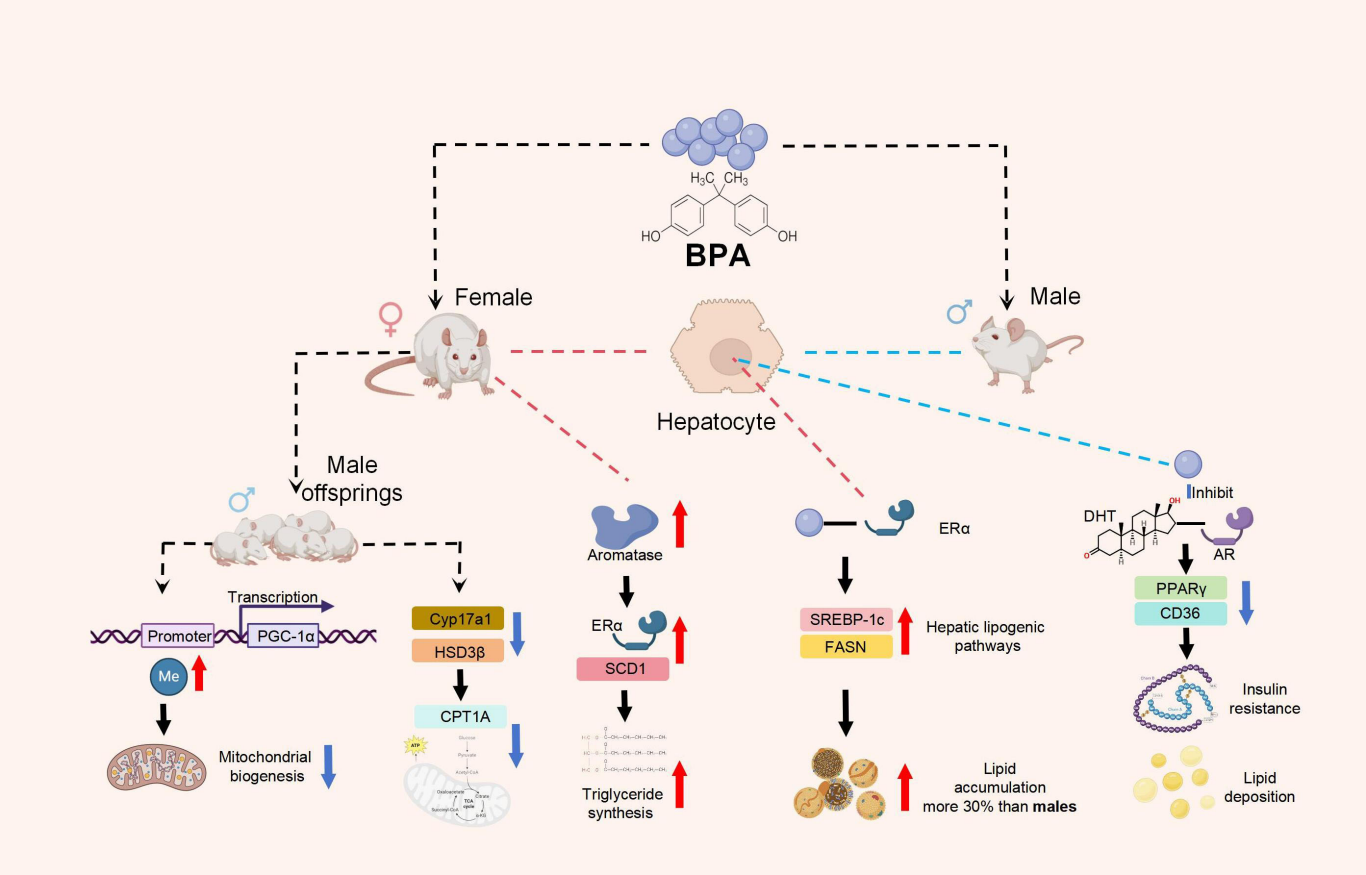
Significant reasons for the sexual disparity of BPA-induced hepatic metabolic dysregulation.

Herein, we summarized three significant explanations for the sexual disparity of BPs-induced hepatic metabolic dysregulation: hormonal receptor modulation, sex hormone interactions, and epigenetic and developmental drivers (**Figure 4**).

BPs exhibit sex-specific hepatotoxicity through divergent interactions with nuclear hormone receptors. Importantly, effects of EDCs like bisphenols may rely on the hormonal status of individuals. Precisely in females, the estrogeno-mimetic effect of EDCs such as BPA may trigger opposite effects as shown in a previous study (89).

In female rodents, BPA preferentially binds to estrogen receptor-α (ERα), activating hepatic lipogenic pathways (e.g., SREBP-1c, FASN) and exacerbating lipid accumulation by up to 30% compared to males (9). Conversely, male susceptibility centers on androgen receptor (AR) disruption: BPA competitively inhibits dihydrotestosterone (DHT) binding, impairing AR-mediated suppression of PPARγ and CD36 expression, thereby driving ectopic lipid deposition and insulin resistance (90–92). Human studies corroborate this dimorphism—urinary BPA levels correlate more strongly with elevated ALT/AST and NAFLD prevalence than females.

BPs dysregulate steroidogenesis, amplifying metabolic risks. Prenatal BPA exposure reduces fetal testosterone synthesis in males by suppressing Cyp17a1 and HSD3β activity, leading to persistent deficits in mitochondrial β-oxidation (CPT1A↓) and hepatic steatosis in adulthood (93). BPA elevates estradiol (E2) via aromatase induction in females, activating ERα-driven SCD1 expression and triglyceride synthesis (94). Paradoxically, postmenopausal females exhibit exacerbated BP sensitivity due to the loss of endogenous E2’s protective effects on lipid homeostasis.

Sex-specific DNA methylation patterns in metabolic genes emerge after developmental BP exposures (95). Male offspring show hypermethylation of PGC-1α promoters, suppressing mitochondrial biogenesis, while females retain demethylated ERβ loci that mitigate lipid peroxidation (96). These epigenetic imprints may explain why childhood BP exposure accelerates NAFLD onset by 5–10 years in males versus females.

### 5.2 Developmental programming of NAFLD

Developmental exposure to BPs during critical windows (gestation, lactation, puberty) induces persistent metabolic reprogramming, predisposing offspring to NAFLD. Prenatal BPA exposure (25 μg/kg/day) in mice triggers hepatic lipogenic gene hypomethylation enhancing lipid synthesis via Nrf2-mediated transcriptional activation (50). Sex-specific susceptibility emerges, with male offspring exhibiting amplified steatosis due to estrogen receptor antagonism and impaired PPARα signaling. Epigenetic modifications in lipid oxidation genes (CPT1A, ACOX1) and gut microbiota dysbiosis persist into adulthood, creating a “second hit” for NAFLD progression (93). Transgenerational effects occur via germline epigenetic inheritance, with F2 progeny showing elevated liver triglycerides despite no direct exposure. These findings underscore developmental BP exposure as a latent driver of metabolic liver disease across lifespans.

### 5.3 Mechanistic insights from model organisms

Sex-divergent responses to bisphenols are rooted in hormone-signaling crosstalk. In male C57BL/6 mice, BPA activates hepatic ERα/PPARγ pathways, amplifying lipogenesis, while females exhibit compensatory estrogen-mediated suppression of SREBP-1c via GPER1. Developmental exposure models reveal transgenerational impacts: prenatal BPA (50 μg/kg/day) in CD1 mice induces hypomethylation of Lxrα promoters in F1 males, priming lipid accumulation through enhanced ChREBP recruitment (44). Zebrafish studies demonstrate AR-mediated susceptibility, where BPS upregulates PPARβ in males but not females, correlating with sex-specific steatosis. Organoid models further highlight sex-dimorphic NLRP3 activation, with testosterone potentiating BPA-induced inflammasome priming in hepatocytes (11). These findings underscore ER/AR interplay and epigenetic reprogramming as key drivers of sex- and developmental-stage-specific vulnerabilities.

## 6 Interactions with other environmental factors

### 6.1 Co-Exposure with chemical pollutants

Nowadays, EDCs are perceived as NAFLD promoters and Di-(2-Ethylhexyl) phthalate (DEHP) and BPA present significant environmental endocrine-disrupting chemical properties (97, 98). DEHP exposure is also associated with the NAFLD risk, based on NHANES database (99). However, the co-exposure of BPA and DEHP has not yet been thoroughly explored. Mechanistically, in Zhang et al.’s recent research, 36 perinatal rats were divided into DEHP (600 mg/kg/d), BPA (80 mg/kg/d), combination, and control groups (100). Screening identified 11 hepatic-damage-related chemical targets. Molecular docking showed strong interactions between 8 metabolic components and PI3K/AKT/FOXO1 pathway targets. Combined exposure induced hepatic steatosis and disrupted glucose/lipid homeostasis via PI3K/AKT/FOXO1-mediated liver dysfunction and insulin resistance.

When tested at non-cytotoxic levels, these substances and BPA-disrupted key regulatory genes govern lipid homeostasis. Specifically, cadmium, PFOA, DDE, and DEHP markedly upregulated DGAT1 (crucial for triglyceride synthesis), while butylparaben enhanced fatty acid transporter FAT/CD36 expression. BPA conversely suppressed CPT1A, a pivotal gene in fatty acid oxidation. Notably, PFOS, BPS, and dibutyl phthalate showed negligible impacts on lipid droplet formation or lipid metabolism-associated genes. Across tested EDCs, lipid accumulation demonstrated positive correlations with SREBF1, DGAT1, and CPT1A expression profiles. These results strengthen evidence linking EDC exposure to NAFLD pathogenesis while demonstrating the value of *in vitro* approaches for identifying environmental contaminants with steatogenic potential and metabolic disruption capabilities.

### 6.2 Environmental modulators of BP toxicity

Environmental co-exposures significantly amplify bisphenol-induced hepatotoxicity through synergistic or additive mechanisms. Phthalates, commonly coexisting with BPs in plastics, exacerbate hepatic lipid accumulation by competitively inhibiting PPAR-α-mediated fatty acid oxidation while amplifying ER stress via the PERK-CHOP pathway. Heavy metals like cadmium and lead act as co-modulators by depleting glutathione reserves, compounding BP-induced oxidative DNA damage in hepatocytes. Particulate matter (especially PM2.5) enhances BP bioaccumulation in liver tissue through AhR-mediated CYP450 inhibition, while microplastics serve as BP transport vectors, increasing intestinal absorption and hepatic bioavailability by 40-60% (83, 101, 102). Dietary factors critically modulate toxicity thresholds: high-fat diets upregulate hepatic CD36 expression, facilitating BP-triggered lipid uptake, whereas polyphenol-rich diets mitigate steatosis via Nrf2 activation. Crucially, circadian disruption from light pollution dysregulates hepatic clock genes (e.g., Bmal1), potentiating BPs-induced metabolic dysfunction. These interactions underscore the necessity for cumulative risk assessments in environmental health policies. Regarding dietary factors, several researches showed that environmental pollutants and high-fat diets affect common metabolic pathways, e.g., modifications in lipid homeostasis (103).

### 6.3 Lifestyle and dietary co-drivers

Concerningly, compared to the low-BMI population, those fatty individuals more easily suffer from NAFLD under BPA exposure. Ribeiro’s group demonstrated that BPA exposure in ovariectomized mice on high-fat diet exacerbated hepatic steatosis, increasing collagen deposition and altering lipid metabolism genes: downregulating Mttp while upregulating β-oxidation (Acox1, Acadvl), mitochondrial uncoupling (Ucp2), ER stress (Hyou1, Atf6), and liver injury markers (Tgfb1, Casp8). BPA caused mild steatosis in normal-diet mice through upregulated lipogenesis (Srebf1, Scd1) and ER stress genes (104). BPA exacerbated HFD-mediated dysregulation of critical regulators in glucose and lipid metabolism, promoted hepatic triglyceride deposition, and aggravated mitochondrial dysfunction through elevating oxidative stress while diminishing cellular antioxidant capacity (73). Perinatal exposure to BPA/experimental diets alters offspring metabolic health (not NAFLD), with maternal phenotype changes driving health trajectories, underscoring maternal diet’s role in environmental exposure assessments (105).

Similar to BPA, according to Xie’s study, BPS exposure (50/500 μg/kg/day) exacerbated HFD-induced NAFLD in mice, increasing liver/body weight ratio, serum ALT/AST levels, and hepatic lipid accumulation. It dysregulated fatty acid metabolism genes (Cd36, Pparγ, Scd-1, Fasn, Pparα) and pro-inflammatory cytokines (TNFα, IL-1β, IL-6) (106). BPS triggered hepatic ferroptosis via altered GPX4, xCT, FTH, and ACSL4 expression, accompanied by ROS overproduction, mitochondrial dysfunction, lipid peroxidation, and GSH depletion - all rescued by ferrostatin-1. Mechanistically, BPS upregulated HMGCS2 in hepatocytes, and HMGCS2 knockdown reversed ferroptosis markers, demonstrating that HMGCS2-mediated ferroptosis drives BPS-aggravated NAFLD.

Lv’s study assessed BPF effects (100 μg/kg) on glucose metabolism in mice fed normal (ND) or HFD. BPF improved glucose metabolism in HFD mice but not ND mice, enhancing insulin signaling in skeletal muscle and elevating liver metabolites linked to carbohydrate digestion and TCA cycle (107). Sustained environmentally relevant BPF exposure enhanced insulin sensitivity and glucose regulation in HFD mice. Thus, BPF also deteriorates the glucose metabolism of HFD mice, potentially contributing to the development of NAFLD.

Despite the limitation of mechanistic evidence in humans, substantially decreasing the fatty diet intake can be a promising method to avoid BPs-induced NAFLD.

### 6.4 Mitigating combined exposures

Addressing synergistic hepatotoxicity from bisphenol co-exposure with phthalates or dietary stressors requires integrated strategies. Regulatory frameworks should adopt cumulative risk assessment models for chemical mixtures, replacing single-pollutant paradigms. Emerging technologies like nanomaterial-based biosensors enable real-time detection of multiple contaminants in food and household products, empowering consumer choices. Public health initiatives must promote BP-free alternatives (e.g., plant-based food coatings, ceramic containers) while discouraging concurrent high-fat diets through nutritional education (108). Pharmacological interventions targeting shared pathways—such as NLRP3 inflammasome inhibitors or PPAR-γ antagonists—may counteract synergistic metabolic disruption (49, 109). Crucially, microbiome-modulating prebiotics (e.g., resistant starch, polyphenols) can mitigate gut-liver axis perturbations amplified by combined exposures (13). Policy reforms mandating eco-design principles for plastics and stricter labeling of “BPA-free” substitutes are essential to break exposure cycles.

## 7 Therapeutic and preventive strategies

### 7.1 Lifestyle intervention

Key lifestyle strategies combine BP exposure reduction, dietary modifications, and developmental-stage-specific protection: (1) Exposure minimization: avoid canned foods, plastic-packaged items, and thermal receipts; prioritize glass/ceramic containers (1, 108). (2) Antioxidant-rich diet: consume polyphenol-rich whole foods (fruits/vegetables) to counteract BP-induced oxidative stress (5). (3) Gut-liver axis support: increase resistant starch (whole grains) and prebiotic fiber to mitigate BP-disrupted gut microbiota (13). (4) Weight management: achieve 5–10% weight loss via calorie-restricted diets and combined aerobic/resistance exercise to reverse hepatic steatosis (3, 107). (5) Implement strict BP avoidance in children/adolescents through school nutrition programs and BP-free lunch packaging, targeting vulnerable metabolic programming windows (110).

### 7.2 Pharmacological approaches

Several researchers recently reported that green tea extract intake improves hepatic steatosis and reduces the development of NAFLD (111–113). Moreover, the detrimental effects triggered by BPA in the pathogenesis of NAFLD can be ameliorated by green tea, Vitamin E, and epigallocatechin gallate (EGCG), the major catechin present in green tea (114). Underlyingly, their antioxidant and anti-inflammatory activity can influence this protective role, regulating lipid metabolism and insulin signaling pathway improvement. Furthermore, Zhang’s results demonstrated that EGCG administration effectively decreased mice’s body weight and liver-to-body weight ratio while simultaneously reducing serum triglyceride and total cholesterol concentrations (115). These metabolic improvements were mechanistically linked to transcriptional regulation, evidenced by suppressed mRNA expression of fatty acid synthesis-related Elov16 and cholesterol biosynthesis-associated CYP4A14, coupled with enhanced fatty acid oxidation-related Lss and cholesterol metabolism-regulating Cyp7a1 genes. Thus, green tea, especially its catechin-EGCG, has a promising application for preventing and treating BPs-induced liver metabolism disturbance.

It has been verified that BPA-induced lipid metabolism dysfunction and pyroptosis are driven by OGT upregulation, which critically stabilizes NLRP3 through direct interaction. Elevated OGT enhances NLRP3 protein stability via enhanced O-GlcNAcylation modification. BPA promotes OGT-mediated O-GlcNAcylation to stabilize NLRP3, accelerating NAFLD progression *in vitro* (49). Thus, targeting the NLRP3-OGT axis may counteract BPA-induced NAFLD pathogenesis.

Interestingly, Xu’s group first reported that oridonin alleviates bisphenol A-induced hepatotoxicity via modulating oxidative stress and metabolic pathways. In rats exposed to BPA (500 mg/kg), oridonin pretreatment (10 mg/kg) significantly reduced serum AST/ALT levels, attenuated hepatic apoptosis, and improved histopathology. UPLC-MS/MS metabolomics identified 28 differential metabolites, indicating oridonin restored BPA-disrupted purine metabolism and phenylalanine/tryptophan biosynthesis (116). Mechanistically, oridonin inhibited xanthine oxidase (XOD) activity and decreased ROS and uric acid levels while increasing hypoxanthine/xanthine content. These findings demonstrate oridonin’s hepatoprotective effects through dual regulation of oxidative stress and metabolic reprogramming.

Resveratrol, a natural polyphenolic compound belonging to the stilbene family, emerges as a promising therapeutic candidate for NAFLD prevention and treatment owing to its combined anti-inflammatory, antioxidant properties, and calorie-restriction-mimicking biological effects (117, 118). Based on Liao’s research, intriguingly, resveratrol butyrate ester (RBE) alleviates BPA-induced liver damage in male offspring rats by modulating gut microbiota (enhancing S24-7/Adlercreutzia abundance), increasing fecal short-chain fatty acids, and activating hepatic Nrf2 pathway to boost antioxidant enzymes (HO-1/SOD/CAT). RBE reinforces intestinal barrier integrity and reduces liver inflammation through gut-liver axis interactions, demonstrating its protective mechanism against developmental metabolic disruption (119).

Gut microbiota metabolites play a pivotal role in NAFLD pathogenesis and therapeutic implications; therefore, probiotic modulation emerges as a novel method for prevention and therapeutic methods (120, 121). Co-administering BPA and SLAB51 (109 CFU/g of body weight; P) restored gut integrity, enriched beneficial microbes, and reduced pathogens. In males, BPA-induced hepatic steatosis/glycogen loss was partially reversed by P, correlating with elevated anserine (neuroprotective) and reduced glutamine in the liver. Females showed no steatosis but heightened energy demand via glycogen depletion; while P reduced hepatic retinoic acid, potentially enhancing BPA detoxification.

### 7.3 Policy and public health measures

Importantly, enforce BPA bans in food/beverage packaging and children’s products, extending EU-style REACH regulations globally. Secondly, research funding for plant-based resins (lignin polymers) and non-estrogenic alternatives should be accelerated through public-private partnerships. National biomonitoring programs should also be established to track urinary BP metabolites and liver enzymes (ALT/AST) in high-risk groups (especially children and factory workers).

## 8 Future directions

### 8.1 Mechanistic research gaps

In **Table 3**, we summarized critical gaps and promising methodologies for BP-Induced NAFLD. A critical knowledge gap lies in understanding the hepatotoxic effects of BP metabolites (e.g., BPA-glucuronide) and their role in transgenerational NAFLD pathogenesis. While parental BPA exposure is linked to offspring metabolic dysfunction, the epigenetic mechanisms—such as DNA methylation, histone modifications, or non-coding RNA regulation—mediating this inheritance remain poorly characterized (122–124). Future studies should delineate how BP metabolites interact with nuclear receptors (e.g., ERα/β) to reprogram hepatic lipid metabolism and oxidative stress pathways across generations.

**Table 3:** Critical research gaps and proposed methodologies for Bisphenol-Induced NAFLD.

Research Direction	Key Questions/Challenges	Proposed Approaches	Significance
Chronic Low-Dose Exposure Effects	Non-monotonic dose-response relationships in real-world exposure scenarios	Longitudinal cohort studies tracking BP levels and liver outcomes over decades	Addresses regulatory gaps in current risk assessment models
Epigenetic Reprogramming	BP-induced DNA methylation changes in PPARγ/SREBP-1c and transgenerational effects	Multi-generational animal studies with epigenome-wide association studies (EWAS)	Explains developmental origins of metabolic dysfunction
Cumulative Exposure Synergy	Interactions between BPs and phthalates/microplastics/high-fat diets	Mixture toxicity studies using factorial design experiments	Explains geographic disparities in NAFLD prevalence
Sex-Specific Susceptibility	Molecular basis for male predominance in BP-associated NAFLD	Sex-stratified analyses in human cohorts + gonadectomized animal models	Guides personalized prevention strategies
Gut-Liver Axis Mechanisms	Role of BP-altered gut microbiota (e.g., TMAO producers) in hepatic inflammation	Metagenomics sequencing combined with fecal microbiota transplantation experiments	Identifies novel therapeutic targets
Alternative BP Toxicology	Long-term safety profiles of BPS/BPF/BPAF substitutes	Comparative toxicokinetic studies using next-gen analogs	Informs safer material development
Developmental Windows of Susceptibility	Critical exposure periods (prenatal vs. perinatal vs. adult)	Time-series exposure models with liver organoids	Guides maternal-child protection policies
Biomarker Discovery	Developing stable biomarkers for cumulative BP exposure assessment	Metabolomics profiling of phase II metabolites + adductomics analysis	Improves exposure quantification in epidemiology
Policy-Relevant Exposure Thresholds	Establishing BP thresholds for hepatotoxicity across populations	Benchmark dose modeling integrated with adverse outcome pathway (AOP) analysis	Supports evidence-based regulatory standards
Intervention Strategies	Efficacy of BP avoidance vs. pharmacological protectants (e.g., antioxidants)	Randomized controlled trials testing combination prevention approaches	Direct translation to clinical practice

Current evidence lacks large-scale, long-term human data correlating BP exposure levels with NAFLD progression. Establishing multinational cohorts (e.g., integrating the NHANES database with Biobanks) is imperative to track temporal changes in gut microbiota composition, circulating BP metabolites, and liver injury biomarkers (e.g., ALT, miRNAs). Such cohorts should stratify populations by sex, age, and metabolic status to identify vulnerable subgroups and dose-response thresholds.

Most research focuses on BPA, yet substitutes like BPS and BPF exhibit similar endocrine-disrupting properties. Their individual and combined hepatotoxicity—particularly in co-exposure with microplastics (e.g., polystyrene)—requires urgent investigation. Mechanistic studies using liver organoids could clarify synergistic disruptions of lipid metabolism genes (e.g., HNF4A, CD36) and epigenetic regulators.

Sex disparities in BPA-induced hepatic steatosis (e.g., female resilience to lipid accumulation) suggest hormonal or genetic modifiers. Comparative analyses of retinoic acid signaling, hypothalamic-pituitary-liver axis interactions, and X chromosome-linked genes may reveal novel therapeutic targets.

### 8.2 Innovative methodologies

Lipid droplets (LDs) critically influence physiological processes, necessitating dynamic visualization in living cells (125). This study utilized QSAR theory to design organelle-targeting carbon dots (CDs), employing Log P values to predict cellular uptake and subcellular localization. Hydrophilic p-phenylenediamine-derived CDs were transformed into lipophilic PA CDs with inherent LD-targeting capability 110 by modifying precursor lipophilicity. These PA CDs successfully tracked LD dynamics and visualized bisphenol A-induced fatty liver disease progression in cellular models. The QSAR-driven strategy demonstrates the potential for developing diverse organelle-specific CDs, offering a robust design framework for subcellular imaging probes.

Human pluripotent stem cell-derived liver organoids (LOs) recapitulate sex- and age-specific responses to BP exposures (126). Co-culturing LOs with gut microbiota-derived metabolites (e.g., short-chain fatty acids) reveals cross-tissue interactions. At the same time, single-cell RNA sequencing uncovers heterogeneous cell subpopulations vulnerable to PS-BPA synergism (127). Machine learning algorithms can integrate LO-based epigenomic (HNF4A methylation), proteomic (CYP2E1/ERα), and lipidomic data to map adverse outcome pathways. Also, CRISPR-edited hepatic organoids can be considered as a drug screening platform for NAFLD (128). Human fetal hepatocyte organoids model NAFLD steatosis under three triggers: fatty acid overload, PNPLA3 I148M mutation, and APOB/MTTP mutations. Drug screening identified steatosis-resolving compounds targeting *de novo* lipogenesis repression (129). Delilah et al. developed FatTracer, a CRISPR platform using APOB−/−/MTTP−/− organoids, screening 35 lipid-related genes. FADS2 emerged as critical enhanced expression increases polyunsaturated fatty acids, suppressing lipogenesis. This system enables mechanistic exploration and therapeutic target discovery for hepatic steatosis.

Deep learning models trained on UPLC-MS/MS metabolomics and LD imaging data can predict structure-hepatotoxicity relationships for emerging BP substitutes. These models, validated through high-content screening in LOs, will accelerate the prioritization of safer alternatives like lignin-based polymers.

### 8.3 Translational priorities

Urgent efforts are needed to validate non-invasive biomarkers for early NAFLD detection in BP-exposed populations. Circulating miRNAs (e.g., miR-122, miR-34a) and epigenetic markers (e.g., HNF4A methylation) show promise for reflecting BPA-induced hepatic steatosis and ERα-mediated lipid dysregulation (127, 130). Concurrently, organoid-derived exosomes could serve as personalized platforms to identify susceptibility biomarkers linked to PS-BPA synergism. Integrating these biomarkers with multi-omics profiles (metabolomics, metagenomics) may enable risk stratification for precision interventions, such as dietary modifications or targeted detoxification therapies.

Harmonizing BP exposure assessments across regions requires establishing international consortia to standardize analytical methods (e.g., quantifying BPA-glucuronide in urine) and NAFLD diagnostic criteria (imaging vs. histology). Leveraging existing cohorts like NHANES and UK Biobank, combined with microplastic exposure mapping, could reveal geographic hotspots of coexposure risks. Priority should be given to vulnerable groups—children, pregnant women, and metabolically compromised individuals—through longitudinal monitoring of gut microbiota shifts and liver enzymes.

While plant-based resins (e.g., lignin derivatives) and non-estrogenic alternatives are emerging, their long-term hepatotoxicity and endocrine-disrupting potential remain unverified. Accelerated safety assessments using liver organoid models and QSAR-driven carbon dot probes (e.g., PA CDs) are critical to avoid regrettable substitutions. Regulatory agencies must adopt adverse outcome pathways (AOPs) for BP analogs, prioritizing compounds with minimal lipid droplet accumulation and CYP2E1 disruption in preclinical screens.

Targeted interventions—probiotics to restore BPA-disrupted gut flora or epigenetic modulators (e.g., curcumin) to counteract DNA methylation changes—should be tested in high-risk communities. Public health policies must integrate BP exposure reduction (e.g., food packaging reforms) with NAFLD prevention programs, emphasizing sex-specific vulnerabilities identified in mechanistic studies.
